# Perianal Hidradenoma Papilliferum Mimicking a Chronic Hemorrhoid: A Case Report

**DOI:** 10.1155/carm/6733314

**Published:** 2026-09-15

**Authors:** Cesar Ramon Razuri Bustamante, Sofía Leonor Prado Cucho, Laura Elizabeth Quiñones Valverde, Angel Luigui Hurtado Vila, Alexis Narvaez Rojas, Gabriel De La Cruz Ku

**Affiliations:** ^1^ Clinica San Pablo, Lima, Peru; ^2^ Universidad Nacional Mayor de San Marcos, Lima, Peru, unmsm.edu.pe; ^3^ Facultad de Medicina, Universidad Cayetano Heredia, Lima, Peru; ^4^ Patología-Oncológica, Laboratorio Qualab, Lima, Peru; ^5^ Universidad Federico Villarreal, Lima, Peru; ^6^ Gastroenterología, Clínica SANNA, Lima, Peru; ^7^ Department of Oncology, Hospital Alejandro Davila Bolanos, Managua, Nicaragua; ^8^ Universidad Cientifica Del Sur, Lima, Peru, ucsur.edu.pe

**Keywords:** apocrine glands, benign tumors, hidradenoma papilliferum, perianal region, surgical pathology

## Abstract

Hidradenoma papilliferum (HP) is a rare, benign neoplasm classically originating from apocrine or mammary‐like glands. While predominantly presenting in the vulvar region, its occurrence in the perianal location is exceptionally rare and frequently misdiagnosed as hemorrhoidal disease or other localized benign lesions. We present the case of a 50‐year‐old female patient reporting acute perianal pain following defecation straining, accompanied by the sensation of an asymptomatic perianal mass present for three years. Clinical examination identified a 1.5 cm well‐circumscribed, reddish‐brown nodule at the anal verge. Excisional biopsy was performed under local anesthesia with 1 cm margins, allowing the wound to heal by secondary intention. Histopathological evaluation confirmed perianal HP, characterized by an arboriform papillary architecture, a double‐layered epithelium, and classic apocrine decapitation secretion without cytological atypia. Complete surgical excision remains the definitive, curative treatment. This case highlights the absolute necessity of histopathological evaluation for atypical, therapy‐resistant perianal lesions to avoid diagnostic anchoring and to definitively rule out malignancy.

## 1. Introduction

Hidradenoma papilliferum (HP) is an uncommon, benign, cystic, and papillary neoplasm. While classically considered to originate from apocrine sweat glands, contemporary histopathological evidence increasingly suggests a derivation from mammary‐like glands situated along the female anogenital milk lines [1]. This entity almost exclusively affects women of reproductive age. The overwhelming majority of HP lesions occur in the vulvar region; presentation in the perianal area is exceedingly rare, occurring approximately four times less frequently than vulvar manifestations [2, 3].

What makes this case unique is the diagnostic challenge it presents to colorectal surgeons and proctologists. Clinically, perianal HP appears as a solitary, well‐demarcated nodule. Due to macroscopic similarities, particularly when localized engorgement or microtrauma occurs, perianal HP is frequently misdiagnosed as thrombosed hemorrhoidal disease, perianal abscesses, or viral verrucous lesions. Consequently, this report details the clinical and histopathological features of an atypical perianal HP to reinforce its critical inclusion in the differential diagnosis of anorectal masses and to outline the definitive surgical management strategy.

## 2. Case Description

A 50‐year‐old female patient presented to the surgical outpatient clinic with a chief complaint of localized perianal pain. The patient’s medical, family, and psychosocial history were unremarkable. Her relevant past interventions included only an ovarian cystectomy on an unspecified date, with no history of prior anorectal procedures or gastrointestinal malignancies.

The patient reported the sensation of an asymptomatic perianal nodule that had been present for approximately 3 years. One month prior to consultation, following significant straining during defecation, the lesion suddenly became painful. She denied active rectal bleeding, pruritus, purulent discharge, or any discernible increase in the mass’s size over the preceding 3 years.

**FIGURE 1:**
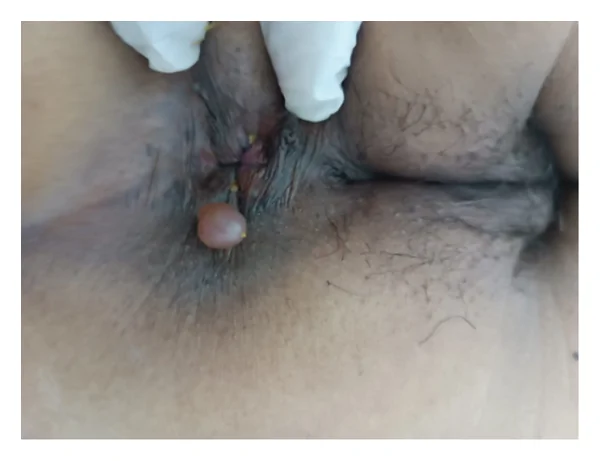
Preoperative clinical appearance of the perianal lesion. A well‐circumscribed nodule with a smooth surface and reddish‐brown coloration is observed in the perianal region.

Physical examination revealed a well‐circumscribed, reddish‐brown nodule measuring approximately 1.5 cm in diameter located at the anal verge (Figure 1). The lesion exhibited a soft‐elastic consistency and a smooth surface and was mobile without tethering to deeper underlying anatomical planes. Palpation elicited mild localized tenderness. Digital rectal examination revealed no intraluminal extensions, sphincter abnormalities, or palpable mucosal defects. No inguinal lymphadenopathy was detected.

The primary diagnostic challenge in this case was distinguishing the lesion from common proctological conditions. The preliminary clinical diagnosis heavily favored a thrombosed external hemorrhoid due to the macroscopic appearance and acute onset of pain following straining [4]. However, the chronic 3‐year history of the nodule raised suspicion for a neoplastic process, necessitating tissue evaluation.

**FIGURE 2:**
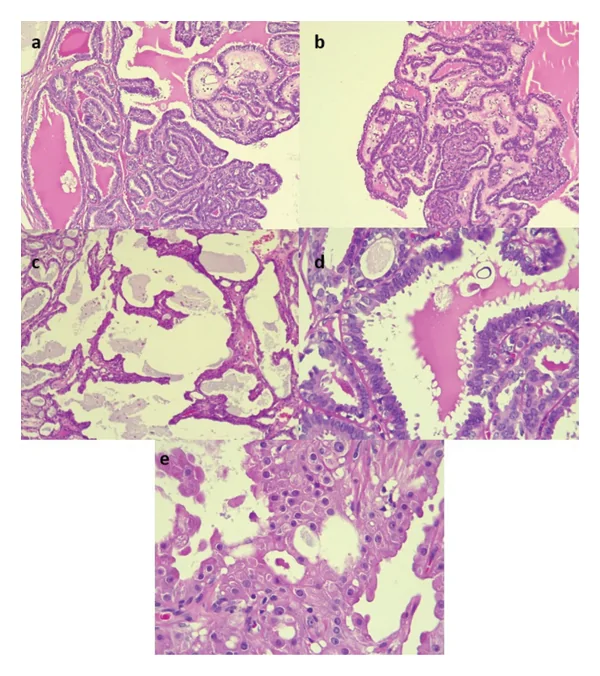
Histopathologic features of perianal hidradenoma papilliferum. (a) Low‐power H&E image (10×) demonstrating the characteristic papillary architecture with fibrovascular cores lined by luminal and myoepithelial cell layers, together with cystic spaces containing eosinophilic material. (b) Low‐power H&E image (10×) showing additional areas of papillary architecture with fibrovascular cores containing fibrous and edematous stroma. (c) H&E image (10×) demonstrating cystic spaces lined by cells with abundant eosinophilic cytoplasm, consistent with oxyphilic metaplasia. (d) High‐power H&E image (40×) demonstrating the characteristic dual cell population, composed of cuboidal‐to‐columnar luminal cells showing secretion toward the lumen and underlying flattened, cuboidal, or ovoid myoepithelial cells. No significant nuclear pleomorphism, frequent or atypical mitoses, or other malignant cytologic features are identified. (e) High‐power H&E image (40×) demonstrating oxyphilic metaplasia characterized by cells with abundant eosinophilic cytoplasm and mildly prominent nucleoli, without malignant cytologic features.

Following excision, macroscopic pathological evaluation revealed a partially cystic, solid soft‐tissue architecture. Microscopic examination identified an arboriform pattern of tubular and papillary structures lined by a characteristic double cellular layer: basal cuboidal myoepithelial cells and luminal columnar cells exhibiting active apocrine decapitation secretion. At low magnification, the lesion demonstrated characteristic papillary and cystic architecture with fibrovascular cores, including areas of fibrous and edematous stroma and focal oxyphilic metaplasia (Figure 2a–c). At higher magnification, the dual luminal and myoepithelial cell layers and apocrine secretion were readily identified, with additional areas of oxyphilic metaplasia (Figure 2d,e). The definitive absence of cytological atypia, elevated mitotic activity, or necrosis supported the benign diagnosis of HP, definitively ruling out squamous cell carcinoma or anal adenocarcinoma.

Surgical excision was pursued for both definitive diagnosis and symptomatic relief. Under local anesthesia, the nodule was entirely excised utilizing a cold scalpel, securing 1 cm peripheral margins to ensure total extirpation without compromising anal sphincter integrity. Hemostasis was achieved via electrocautery. The surgical defect was deliberately left open to heal by secondary intention to mitigate the high risk of infection inherent to the perianal region.

**FIGURE 3:**
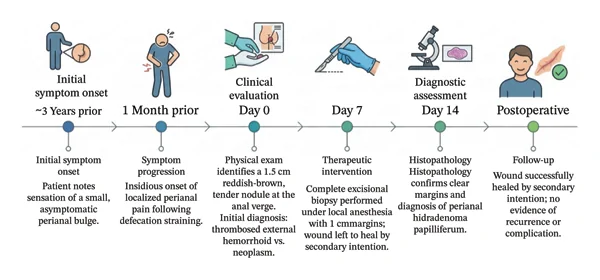
Timeline of the patient’s episode of care, highlighting clinical presentation, intervention, and outcomes.

The patient’s postoperative course was favorable, and intervention tolerability was high. Healing by secondary intention progressed without adverse or unanticipated events, such as surgical site infection or dehiscence. At the most recent clinical follow‐up, the patient was asymptomatic with no evidence of local recurrence, signifying an excellent clinician‐assessed and patient‐assessed outcome. The complete clinical course is summarized in Figure 3.

## 3. Discussion

A primary strength of our approach to this case was the immediate utilization of an excisional biopsy rather than empirical observational management, allowing for simultaneous definitive diagnosis and curative therapy. A limitation of this case is the lack of an extensive immunohistochemical (IHC) panel. However, the characteristic histomorphologic features on routine H&E examination supported the diagnosis of HP. In lesions with classic morphology and no cytologic atypia, increased mitotic activity, or necrosis, extensive IHC may not be routinely required, although it remains valuable in atypical or diagnostically challenging cases.

The presentation of HP in the perianal region represents a highly atypical anatomical variant. The indolent, multiyear evolution of the nodule supports the strictly benign nature of the lesion, whereas the acute onset of pain likely resulted from localized microthrombosis within the fibrovascular stroma during defecation straining. Recent advancements in immunohistochemistry have solidified the mammary‐like gland origin of HP. These lesions characteristically exhibit a GATA3, estrogen (ER), and progesterone (PR) positive profile, frequently harboring PIK3CA mutations [5]. Furthermore, novel markers such as TRPS1 have demonstrated highly sensitive, diffuse nuclear expression in these anogenital mammary‐like gland lesions, proving comparable to GATA3 [6]. When morphology is equivocal, IHC can further aid in distinguishing HP from its histologic mimickers. Luminal cells typically express GATA3, ER, PR, and androgen receptor (AR), whereas the peripheral myoepithelial layer can be highlighted by markers such as p63, smooth muscle actin (SMA), and CK14. PAX8 is typically negative, providing additional support in the differential diagnosis of glandular lesions [5]. This biphasic immunophenotype can be particularly useful in morphologically atypical or diagnostically challenging cases [7].

**FIGURE 4:**
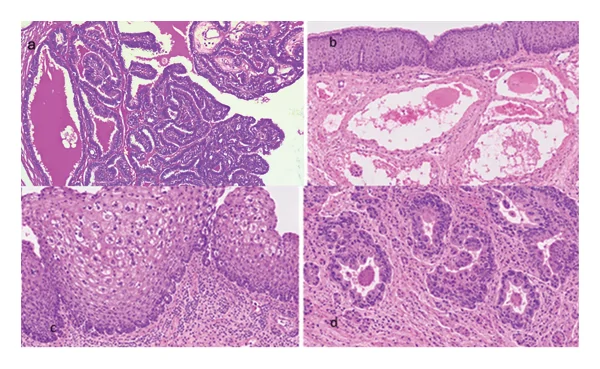
Histopathologic differential diagnoses of perianal hidradenoma papilliferum. Representative H&E images of important differential diagnoses. (a) Fibroepithelial anal polyp, demonstrating hyperplastic mucosal changes, mixed inflammation, and vascular structures within a fibrovascular core. (b) Hemorrhoid, characterized by dilated and congested venous plexuses within the lamina propria, without epithelial dysplasia or malignancy. (c) Condyloma, demonstrating hyperkeratosis, epithelial hyperplasia, parakeratosis, hypergranulosis, and changes associated with human papillomavirus infection. (d) Adenocarcinoma, demonstrating infiltration of the lamina propria by atypical glands with nuclear enlargement, hyperchromasia, mitotic activity, and nuclear pleomorphism.

Perianal HP must be integrated into the differential diagnosis of nodular anorectal lesions. Important histopathologic mimickers include fibroepithelial polyps, hemorrhoids, condylomas, and malignant glandular lesions such as adenocarcinoma (Figure 4). These entities demonstrate distinct histomorphologic features that can generally be distinguished from the characteristic papillary, cystic, and biphasic architecture of HP on routine H&E examination. Due to its macroscopic mimicry of thrombosed hemorrhoids, rigorous histopathological analysis via complete local excision with adequate margins remains the gold standard, ensuring an accurate diagnosis, preventing recurrence, and definitively ruling out malignancy.

## Author Contributions

We are accepting full responsibility for the conduct of the study. Gabriel De la Cruz Ku and Alexis Narvaez Rojas provided clinical feedback and wrote the report.

## Funding

The authors declare that no financial support was received for the research, authorship, and/or publication of this article.

## Ethics Statement

The authors declare that this case report adheres to the ethical principles outlined in the Declaration of Helsinki for medical research involving human subjects.

## Consent

Written informed consent was obtained from the patient for the publication of any potentially identifiable images or data included in this article.

## Conflicts of Interest

The authors declare no conflicts of interest.

## Patient Perspective

“I had noticed the small lump for a few years, but because it didn’t bother me, I mostly ignored it. When it suddenly became painful after a difficult trip to the bathroom, I was initially very anxious that it might be something serious or malignant like cancer. My doctor explained that we needed to remove it to be certain. The excision procedure itself was quick and entirely manageable under local anesthesia. While the healing process required daily sitz baths and careful hygiene, I am incredibly relieved that the biopsy confirmed the tumor was completely benign. The pain is entirely gone, and I am very satisfied with the outcome and the care I received.”

## Supporting Information

Additional supporting information can be found online in the Supporting Information section.

## Supporting information


**Supporting Information** CARE‐checklist‐English‐2013.

## Data Availability

The raw data supporting the conclusions of this article will be made available by the authors without undue reservation.
